# The complete chloroplast genome and phylogenetic analysis of *Stewartia sichuanensis* (Theaceae), a Chinese endemic tree with narrow distribution

**DOI:** 10.1080/23802359.2023.2192829

**Published:** 2023-03-29

**Authors:** Han-Yang Lin, Chao-Nan Cai

**Affiliations:** a School of Advanced Study, Taizhou University, Taizhou, China; b Zhejiang Provincial Key Laboratory of Plant Evolutionary Ecology and Conservation, Taizhou University, Taizhou, China

**Keywords:** *Stewartia sichuanensis*, chloroplast genome, endemic plant of China, phylogeny

## Abstract

*Stewartia sichuanensis* is a rare plant species of Theaceae and is endemic to China. Its distribution area is highly restricted, and genomic information is extremely limited. The present study reports the first complete chloroplast of *S. sichuanensis*. The chloroplast genome length was 158,903 bp, with a GC content of 37.3%. The chloroplast genome was comprised of an 87,736 bp long large single copy (LSC), an 18,435 bp long small single copy (SSC), and two copies of inverted repeat (IR) regions of 26,366 bp. It contained 129 genes, including 85 encoding, 36 transfer RNA, and eight ribosomal RNA genes. The phylogenetic analysis suggested that *S. sichuanensis* was closely related to *S. laotica* and *S. pteropetiolata*.

## Introduction

Tea family (Theaceae) members are of great economic and ecological importance (Zhang et al. 2022). One of the genera, *Stewartia* L. shows a disjunct distribution between eastern Asia and eastern North America (referred to as the EA-ENA disjuncts; Wen 1999). Hence, the augmenting of *Stewartia* genomic resources will facilitate a better understanding of the evolutionary history of the EA-ENA disjuncts.

*Stewartia sichuanensis* (S. Z. Yan) J. Li & T. L. Ming shows a narrow distribution exclusively restricted to Pingshan Co., Sichuan province, China (Li 1996; Ming and Bartholomew 2007). According to *Flora of China*, *S. sichuanensis* is an evergreen shrub or small tree with pubescent branchlets and 1–1.5 cm long petioles surrounded by approximately 2 mm wide wings (Ming and Bartholomew 2007). It has 7.5–10 cm × 3–4.5 cm leathery leaves, which are elliptic or obovate-elliptic. With solitary flowers and orbicular sepals, *S. sichuanensis* mostly resemble *S. micrantha* and *S. calcicola* morphologically (Ming and Bartholomew 2007). Therefore, *S. sichuanensis* is a rare plant that can play a critical role in deciphering the diversification trajectory of *Stewartia*. Yet, the genetic information of *S. sichuanensis* remains extremely poor, and the phylogenetic placement of *S. sichuanensis* within *Stewartia* remains elusive.

In this study, we report the first complete chloroplast genome of *S. sichuanensis* (NCBI GenBank accession number: ON853833) and reveal its phylogenetic relationships to other *Stewartia* species.

## Materials and methods

**Figure 1.**
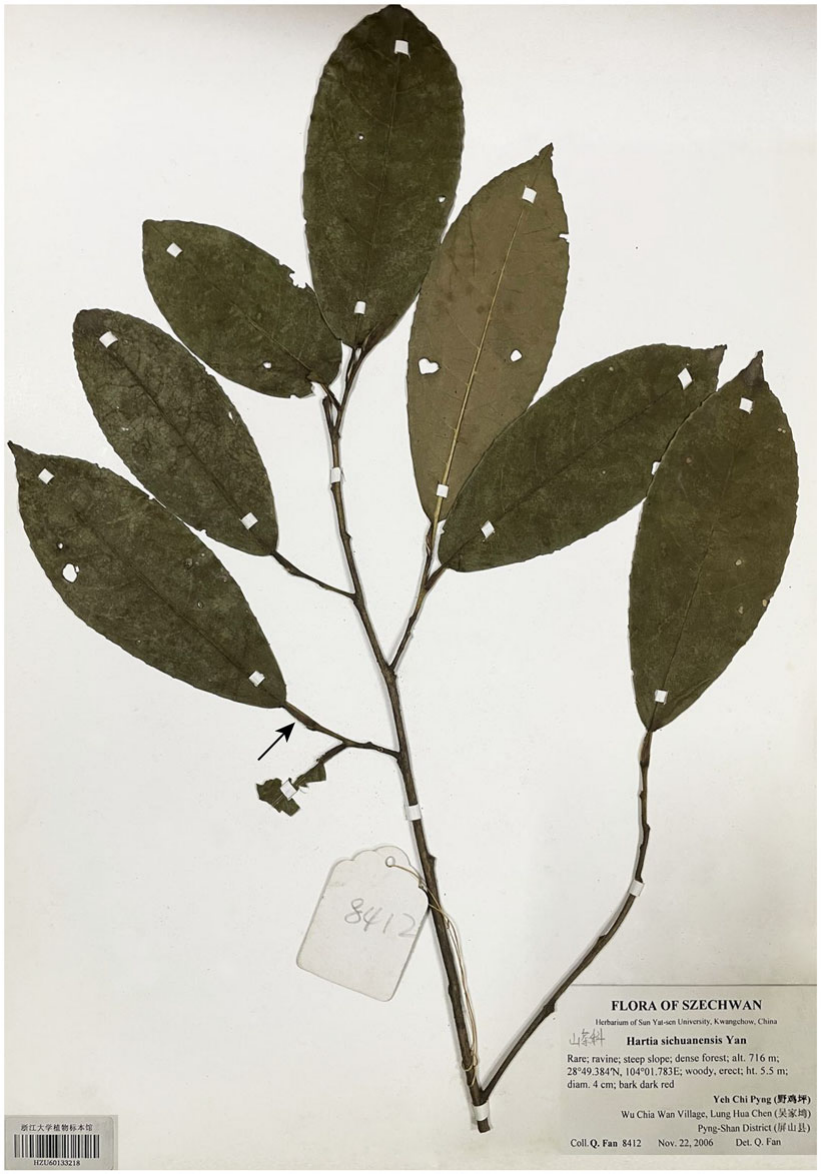
The voucher specimen of collected *Stewartia sichuanensis* (Housed at the Herbarium of Zhejiang University under No. HZU60133218). It shows the typical petiole wing (indicated with a black arrow) and leathery obovate-elliptic leaves of *S. sichuanensis*. Photo courtesy by Han-Yang Lin (Taken on 2022-06-01 at HZU).

Fresh leaf materials were sampled from Wujiawan Village, Pingshan Co., Sichuan, China (28°49′23″N, 104°1′47″E). The voucher specimen (Q. Fan 8412) was deposited at the Herbarium of Zhejiang University (HZU) under No. HZU60133218 (contact person: Han-Yang Lin; email: hylin@zju.edu.cn) (Figure 1).

The total genomic DNA was isolated using DNA Plantzol (Invitrogen, Carlsbad, California, USA) following the manufacturer’s protocol. The high-throughput sequencing was performed using the Illumina HiSeq 2500 platform (Novogene, Tianjin, China). Raw paired-end reads of 150 bp were processed using SOAPnuke (Chen et al. 2018) to remove adapters and low-quality sequences. Then, the complete chloroplast genome was assembled using GetOrganelle (Jin et al. 2020) and was later annotated using CpGAVAS2 (Shi et al. 2019). Schematic maps of the *cis*-splicing genes and *trans*-splicing genes were drawn using CPGView (Liu et al. 2023).

To determine the phylogenetic placement of *S. sichuanensis*, 20 available *Stewartia* chloroplast genomes were obtained from NCBI GenBank (Lin et al. 2019). A consensus maximum-likelihood (ML) phylogenetic tree was constructed using IQ-TREE2 with 1000 ultra-fast bootstrap replicates and the UNREST + FO + I+G4 DNA substitution model [iqtree2 -s XXX.fasta -B 1000 -alrt 1000] (Kalyaanamoorthy et al. 2017; Hoang et al. 2018; Minh et al. 2020).

## Results

**Figure 2.**
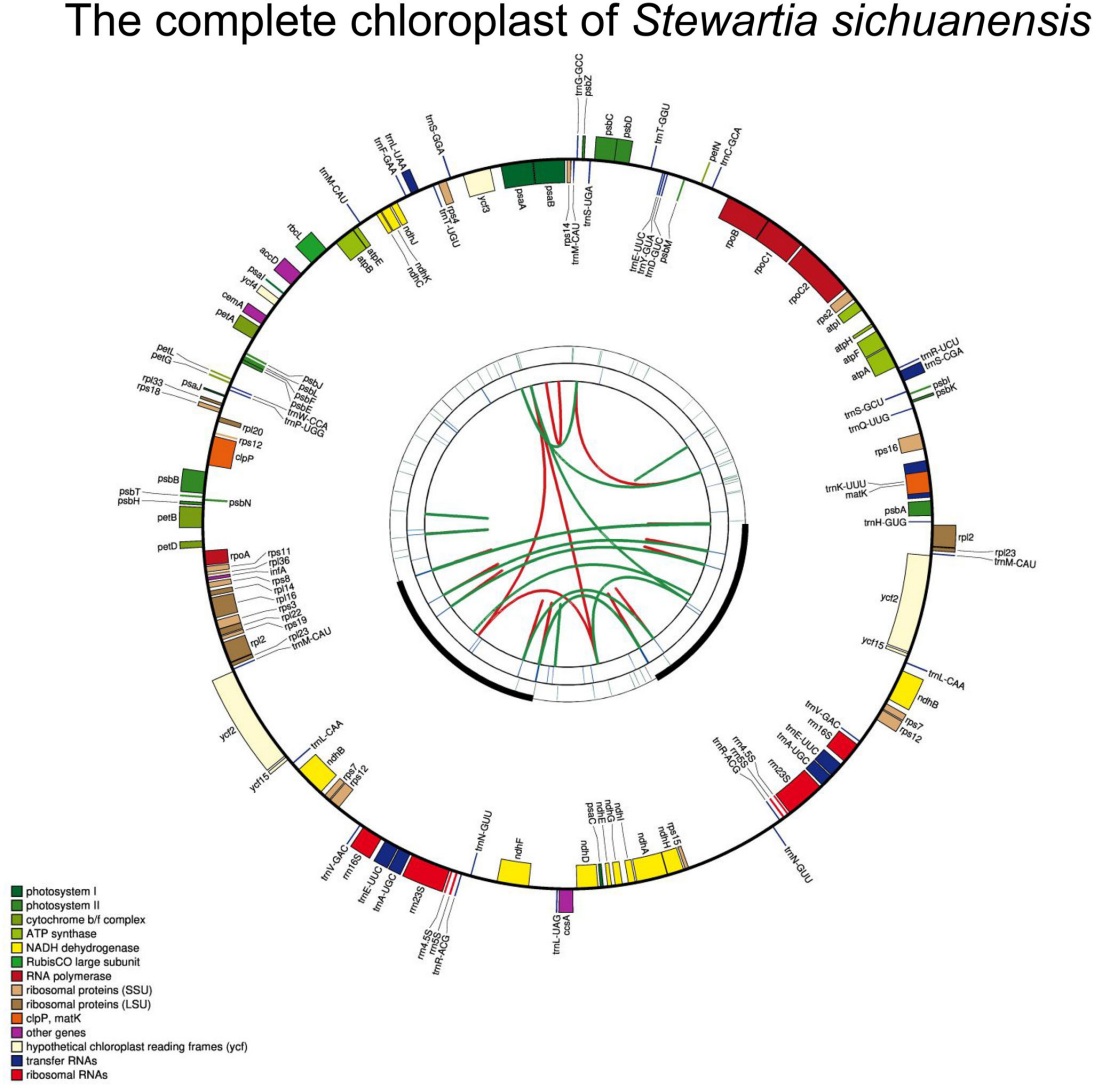
Schematic representation of the *Stewartia sichuanensis* chloroplast genome generated by CpGAVAS2. The map contains four rings. From the center outwards, the first circle shows the forward and reverse repeats connected with red and green curves respectively. The next circle shows the tandem repeats. The third circle shows the microsatellite sequences identified by MISA. Between the third and the fourth circle, two inverted repeat (IR) regions are marked with bold arcs. The fourth circle shows the gene structure on the plastome. The genes were colored based on their functional categories.

**Figure 3.**
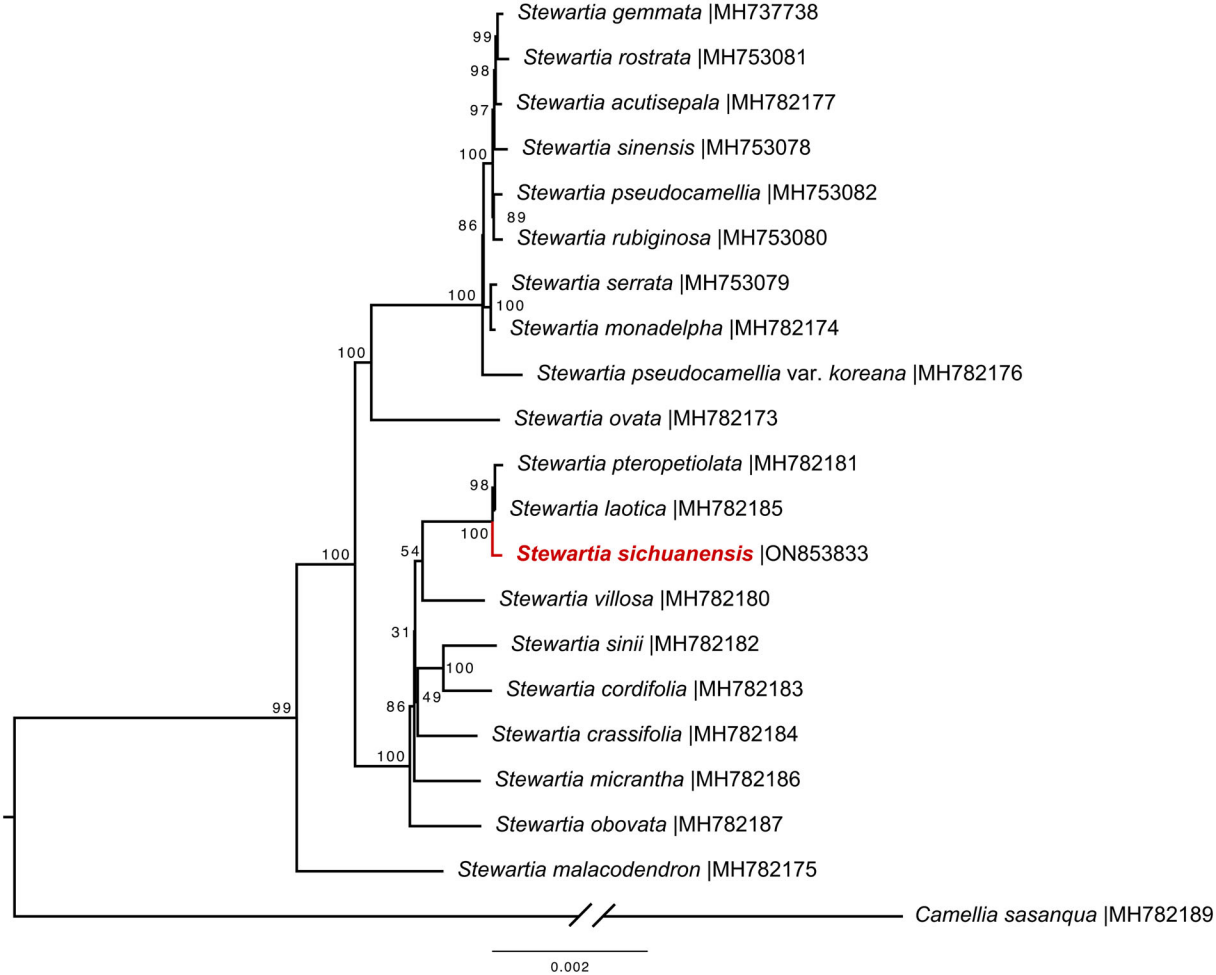
The consensus maximum-likelihood (ML) phylogenetic tree of 20 *Stewartia* species with *Camellia sasanqua* designated as the outgroup based on complete chloroplast genome sequences. All sequences have been published by Lin et al. (2019) except the chloroplast genome of *S. sichuanensis.* The ML analysis was performed using IQ-TREE2 with 1000 ultra-fast bootstrap replicates. The value of bootstrap support (%) of each clade is shown. *S. sichuanensis* (highlighted red) is closely related to *S. laotica* and *S. pteropetiolata* (100% bootstrap support).

After the quality control, we obtained 3.11 Gb of clean sequencing data. The length of the assembled *S. sichuanensis* complete chloroplast genome was 158,903 bp, with a GC content of 37.3%. The read coverage depth is sufficient (with an average of 80 ×), indicating the robustness of genome assembly (Supplementary Figure 1). The chloroplast genome showed a conserved circular structure comprising a large single copy (LSC) of 87,736 bp, a small single copy (SSC) of 18,435 bp, and two copies of inverted repeat (IR) regions of 26,366 bp. The complete chloroplast genome contained 129 genes, including 85 encoding, 36 transfer RNA, and eight ribosomal RNA genes (Figure 2). The genome contained the 13 *cis*-splicing genes and one *trans*-splicing gene (Supplementary Figures 2 and 3). The constructed *Stewartia* phylogeny strongly supported that *S. sichuanensis* was most affinitive to *S. laotica* and *S. pteropetiolata* (Figure 3).

## Discussion and conclusion

The constructed *Stewartia* phylogeny was well-resolved and is highly consistent with a previous report based on plastomic data (Lin et al. 2019). It is the first time that the phylogenetic position of *S. sichuanensis* being unveiled. It was suggested that *S. laotica* is sister to *S. pteropetiolata*, which is congruent with the present results (Lin et al. 2019). To conclude, the release of the chloroplast genome of *S. sichuanensis* provides valuable genetic and phylogenetic information for future research focusing on both *Stewartia* and the EA-ENA disjuncts.

## Supplementary Material

Supplemental Material

## Data Availability

The genome sequence data that support the findings of this study are openly available in GenBank of NCBI at (https://www.ncbi.nlm.nih.gov/) under accession no. ON853833. The associated BioProject, Bio-Sample, and SRA numbers are PRJNA853182, SAMN29363576, and SRR19858886, respectively.
