# Supplementary material for: The complete chloroplast genome and phylogenetic analysis of Stewartia sichuanensis (Theaceae), a Chinese endemic tree with narrow distribution
Source: Mitochondrial DNA B Resour. 2023 Mar 29;8(3):457–60. doi: 10.1080/23802359.2023.2192829 (PMC10062219; doi:10.1080/23802359.2023.2192829)
Supplement: Supplemental Material [file TMDN_A_2192829_SM9088.docx]

**
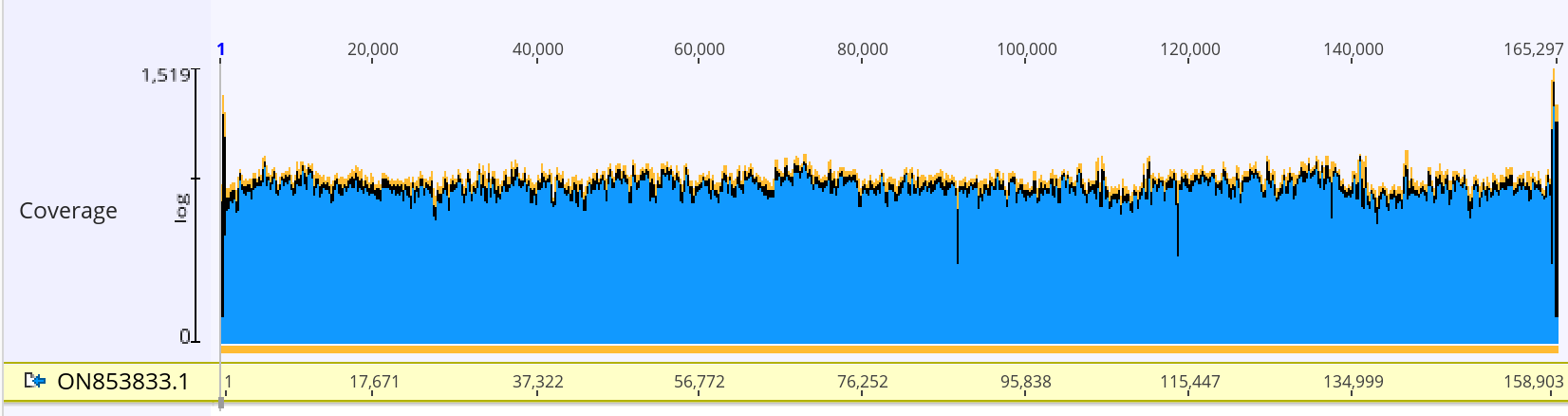
**

**Supplementary Figure 1.** The map of read coverage depth. A total of 80,061 paired reads were mapped to the assembled chloroplast genome of *Stewartia sichuanensis* using Geneious software. The mean read coverage is 80 ×. Read depth exceeding 40 × is highlighted orange.

**Supplementary Figure 2.** The schematic map of the *cis*-splicing genes in the *Stewartia sichuanensis* chloroplast genome. The genes are arranged based on their order in the chloroplast genome. The exons are displayed in black while the introns are displayed in white. The arrow indicates the direction of the gene. The map was drawn by CPGView.

****Supplementary Figure 3.** The schematic map of the *trans*-splicing gene *rps*12 in the *Stewartia sichuanensis* chloroplast genome. It has three unique exons. Two of them are duplicated as they are located in the inverted repeat (IR) regions. The map was drawn by CPGView.
